# Domiciliary high-flow treatment in patients with COPD and chronic hypoxic failure: In whom can we reduce exacerbations and hospitalizations?

**DOI:** 10.1371/journal.pone.0227221

**Published:** 2019-12-30

**Authors:** Ulla Møller Weinreich

**Affiliations:** Department of Respiratory Diseases, Aalborg University Hospital, and The Clinical Institute, Aalborg University, Aalborg, Denmark; National Yang-Ming University, TAIWAN

## Abstract

**Introduction:**

Domiciliary High-flow, humidified, nasal cannula (HFNC) is a possible add-on in patients with chronic respiratory diseases. This post-hoc study investigates benefit of HFNC in subgroups of advanced COPD patients with chronic hypoxic failure on reduction of exacerbations and hospitalizations.

**Methods:**

One hundred patients were randomized to HFNC in a previous trial. Subgroups with 0–1 (N = 32) respectively two or more (2+) (N = 68) exacerbations 12 months pre-study were investigated. Changes in number of exacerbations and hospitalizations pre- and in study were analyzed, corrected for HFNC days with HFNC.

**Results:**

Patients were comparable at baseline. Exacerbations increased in subgroup 0–1 (p = 0.01) and decreased in subgroup 2+ (p = 0.03). Correcting for HFNC days no correlation was seen in subgroup 0–1 (p = 0.08), but in subgroup 2+ (p<0.001). Number of hospitalizations increased in subgroup 0–1 (p = 0.01) with no change in days of hospitalization (p = 0.08). Number and days of hospitalization decreased in subgroup 2+ (p = 0.002 resp. 0.025). Correcting for HFNC days no correlation was found in number or days of hospitalization in subgroup 0–1 (p = 0.48 and p = 0.65). Positive correlation was found in subgroup 2+ (both p<0.001).

**Conclusion:**

In patients with advanced COPD, chronic hypoxic failure and two or more exacerbations per year, HFNC significantly reduced exacerbations and hospitalizations.

## Introduction

Chronic obstructive pulmonary disease (COPD) is globally the single most important respiratory disease; by now the third leading cause of death in the United States [1], expected to become the third leading cause of death in the world by 2020 [2]. In COPD patients, exacerbations are defined as a sustained worsening of a patient’s condition beyond normal day-to-day variations that is acute in onset and require a change in medication and/or hospitalization[3]. When leading to hospitalization exacerbations are considered a severe and are, across all stages of airflow limitations, associated with an increased mortality risk [4]. As such, exacerbations are of major importance as it has a significant and detrimental impact on health status and outcome in these patients and in the GOLD guidelines patients are stratified into different strata depending on number of exacerbations (0–1 or 2+) with in the last 12 months [5]. The strive to reduce exacerbations has therefore been the aim of many COPD research programs in recent years in order to obtain beneficial effect on patient outcomes and prognosis [6]

High-flow, humidified, nasal cannula with optional supplementary oxygen delivery (HFNC) has evolved from being an in-hospital treatment to be a possible add-on for domiciliary use in patients with chronic respiratory diseases [7]. As such, previous studies have shown increased time to first exacerbation and improvement in Forced Expiratory Volume in the first second (FEV1) in patients with obstructive lung diseases and mucus retention challenges [8]. Furthermore, short term studies in patients with stable, advanced COPD and chronic hypoxemic respiratory failure have demonstrated a reduction in respiratory rate [9] and partial in pressure of carbon dioxide in the blood (PaCO_2_) [10] as well as an increased exercise performance [11]. In a long term randomized controlled trial (RCT) in patients with COPD and chronic hypoxemic respiratory failure, hereafter called the Aalborg-study, we recently demonstrated a reduction in number of exacerbations and a reduction in number of hospitalizations when correcting for days of use of HFNC, which was the primary outcome of this study [12].

However, domiciliary HFNC is still in its cradle. We still need to investigate whether all patients benefit equally from HFNC treatment.

The aim of this post hoc analysis on the data from the Aalborg-study [12] is therefore to further examine the primary outcomes of the initial study and investigate whether subgroups of patients, to whom an add-on of HFNC would be of special benefit in terms of reducing number of exacerbations as well as days and number of hospitalizations, can be identified.

## Methods

In the Aalborg study [12] 100 patients with advanced COPD and chronic hypoxic failure (i.e. three arterial blood gases during stable conditions demonstrating hypoxemia[13]) were randomized to usual care plus HFNC for 12 months. The inclusion process and follow up is described in detail in the Aalborg-study [12] and therefore only summarized below.

Patients were to receive long term oxygen therapy (LTOT), prescribed by a Pulmonary Medicine specialist, at least three months prior to study start. Exclusion criteria were malignant disease, terminal non-malignant disease other than COPD, unstable psychiatric disease, home-treatment with non-invasive ventilation. A change of smoking habits during the study period would lead to exclusion. All patients received personalized inhaled medicine according to GOLD guidelines[14], had previously undergone pulmonary rehabilitation, and were in specialized care in connection with LTOT treatment, according to the GOLD recommendations[15]. Change of medication and attending rehabilitation were allowed, if recommended by the patients’ usual caregivers.

Patients were treated with LTOT plus HFNC home treatment delivered by AIRVO^™^ via Optiflow^™^ nasal cannula (both Fisher & Paykel Healthcare, Auckland, New Zealand) at a recommended 20 L/min flowrate, 8 hours per day, preferably at night; however, there were no restrictions in duration of use nor time of day.

Baseline descriptive data on age and gender, lung function, in terms of FEV1 in percent of expected value (FEV1%); smoking status (never smoker/previous (+ 6 months)/ or present smoker); PaCO_2_; dyspnea (modified Medical Research Council (mMRC) score) and walking distance in 6 minute walk test were included in the post hoc analysis. Kolmogorov-Smirnov’s test was applied, and data were normally distributed apart from mMRC score. Data on background information are presented in per cent or as mean and standard deviations (SD). Furthermore, data on number of exacerbations one year pre study as well as number of exacerbations during the study period were registered. Likewise, number and days of hospitalization (ICD10 code J440-9 (COPD) at discharge) one-year pre study and during the study period were included in the analysis. Lastly, days of treatment with HFNC were included.

Patients were divided into subgroups of those with 0–1 exacerbation in the year prior to inclusion and those with 2+ exacerbations in the year prior to inclusion.

Using *Sample-size Calculator* (Clin.calc.com) the sample size for detecting a difference in number of exacerbations in patients with 2+ exacerbations were calculated, based on the mean number of exacerbations at baseline, alpha 0.05 and a power of 0.8. Adequate sample size was calculated to 42 patients.

Descriptive data were provided as means and standard deviations and subgroups compared with paired t-test apart from smoking status, which was compared by chi square test. Number of exacerbations and number and days of hospital admission pre study were also compared by paired t-test between subgroups as were exacerbations, hospital admissions and HFNC days in study. In addition, a one-sample t-test was performed on number of exacerbations; number and days of hospital admission compared to baseline. Paired t-test was also used for comparison in change in exacerbations; days and number of admissions in group 0–1 exacerbations and controls in the RCT, in the appendix.

A Poisson regression test was made to investigate the effect of: PaCO_2_ at baseline; exacerbations and number and days of hospital admissions one year prior to inclusion on the number of exacerbations in study, corrected for days of use of HFNC (HFNC days). As data on number- and days of hospitalization were non-normally distributed an ANOVA test was made to investigate the effect of the above-mentioned parameters on number- and days of hospital admission. The analyses above were repeated on subgroups with 0–1 and 2+ AECOPD prior to inclusion. A minimalized dataset for the paper is accessible at Aalborg University VBN[16]

The study was approved by the North Jutland Ethical Committee (N-20110057), the Danish Data Protection Agency (2008-58-0028) and registered at ClinicalTrials.gov (NCT 02731872). All patients were informed according to the Helsinki Declaration and written consent was obtained prior to inclusion in the study.

## Results

**Fig 1:**
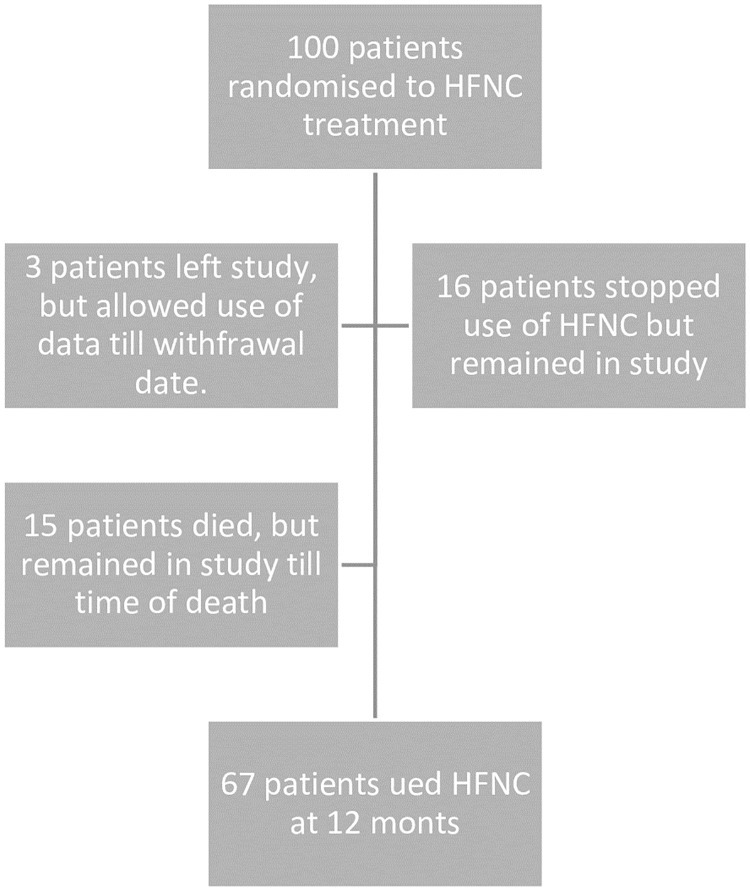
Flow diagram of patients included in the post hoc analysis.

Of the 100 patients were included in this post hoc study 32 patients had 0–1 AECOPD in the year prior to inclusion and 68 patients had 2+ exacerbations. Fig 1 describes the adherence of the patients. Descriptive data are demonstrated in Table 1. Despite a tendency towards patients with 2+ exacerbations being younger, more likely female, with a shorter walking distance there were no significant differences between subgroups (no p-values <0.2). Sixty-seven patients used HFNC throughout the study period; however only three patients left the study entirely and exacerbation.

**Table 1:** Background information on included patients, the entire study population and subgroups. Where nothing else stated data are presented as means (standard deviations).

	All HFNC-treated (N = 100)	0–1 exacerbation in the preceding year (N = 32)	2+ exacerbations in the preceding year (N = 68)
**Gender, % female**	56	50	59
**Age, years**	71(8.2)	74(9)	70(7.6)
**Smoking status, N, never/present/former**	1/14/85	0/5/27	1/9/58
**mMRC-score**	3.3 (0.9)	3.1(0.9)	3.4 (0.9)
PaCO_2_, kPa	6.5 (1.3)	6.6(1.5)	6.4(1.2)
PaO_2_, on LTOT, kPa*	9.9 (1.8)	9.9 (1.7)	9.8 (1.8)
**Current Oxygen flow**	1.6 (0.7)	1.7 (0.6)	9.8 (!.8)
**FEV1%**	29.8 (12.6)	31,1	29.0(12.2)
**6MWT, [N], meters**	[91], 254.6 (89.2)	[28],245(83)	[63],259.2(92.8)
**HFNC HFNC days**	248(141)	257 (131)	241(126)
**HFNC, hours/day**	6.0 (3.0)	6.1(3.4)	6.0 (4.4)

mMRC = modified Medical Research Council Score; PaCO_2_ = arterial partial pressure of carbondiaoxide; PaO_2_ = arterial partial pressure of oxygen; LTOT = Long Term Oxygen treatment; FEV1% = Forced Expiratory Volumen in the first second in % of expected value; 6MWT = six minute walk test *PaO_2_-value on prescribed oxygen

**Fig 2:**
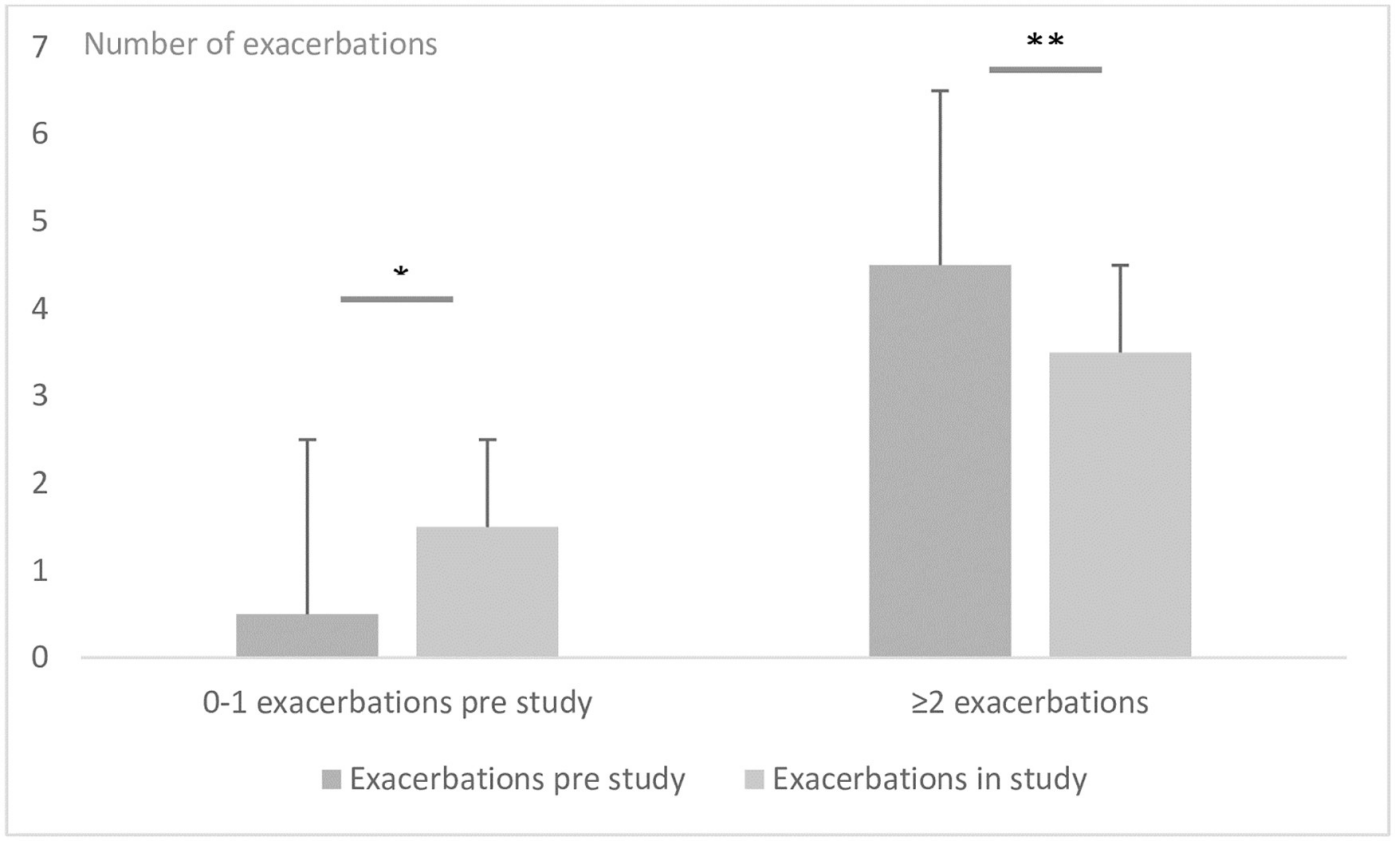
Number of exacerbations 12 months prior to study inclusion and during 12 months’ study participation in subgroups with 0–1 and 2+exacerbations in the year prior to inclusion. *p = 0.01 **p = 0.03.

Fig 2 demonstrates the number of exacerbations in the two subgroups of patients with 0–1 exacerbation and 2+ exacerbations. There was a significant increase in number of exacerbations pre- compared to in study in the group with 0–1 exacerbation pre-study (p = 0.01), and, performing one sample t-test, comparing to baseline, a significant difference was also seen (p = 0.014). A significant reduction in exacerbations pre- compared to in study in patients with 2+ exacerbations (p = 0.03). There was a close to significant difference in the number of AECOPD in study between the two subgroups (p = 0.05).

**Fig 3:**
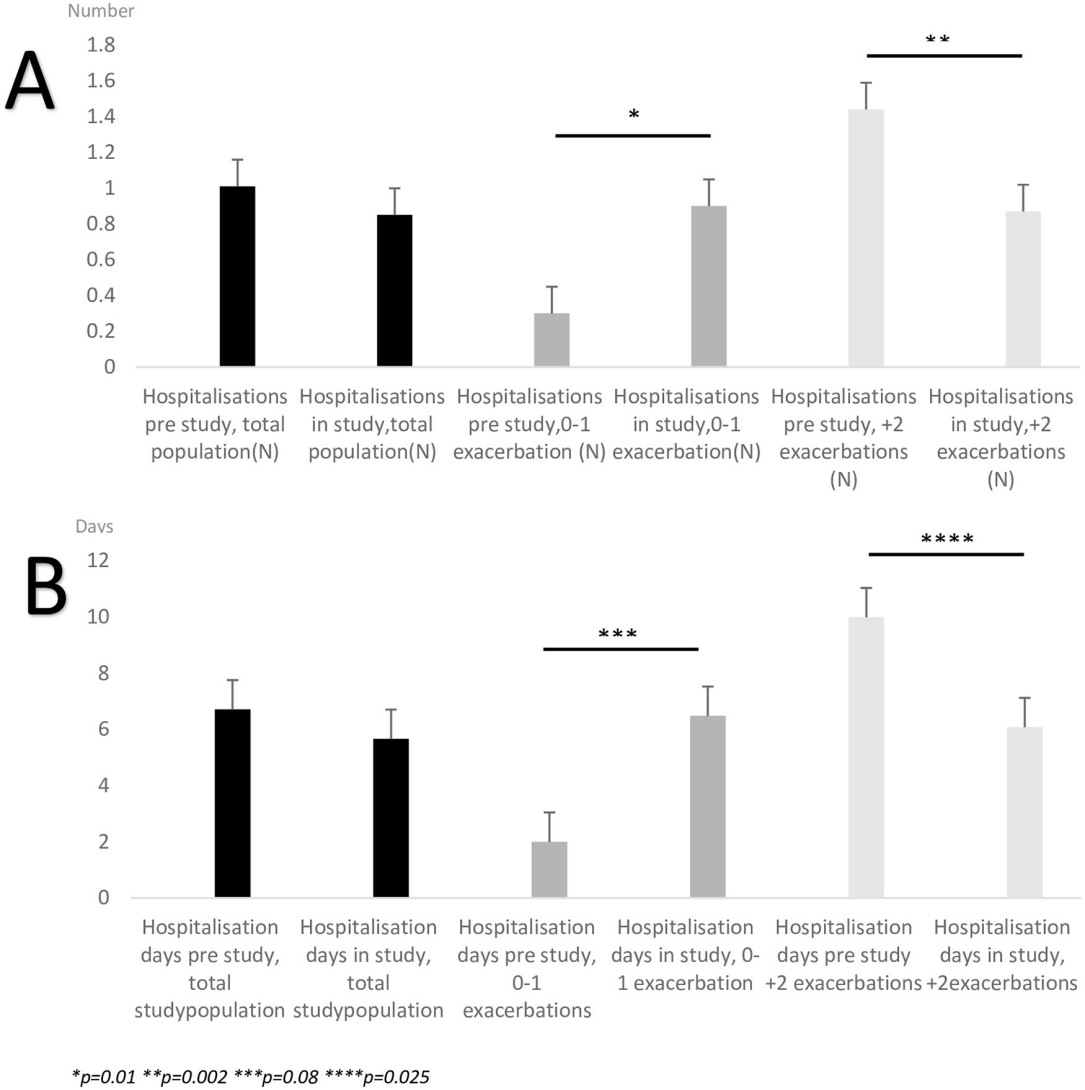
Number of hospitalizations (plate A) and days of hospitalization (plate B) for the total study population and patients with 0–1 exacerbation and 2+ exacerbations the pre-study year.

Fig 3A and 3B demonstrates the number- and days of COPD-related hospitalization in subgroups of patients with 0–1 respectively 2+ exacerbations one year prior to hospitalization and during the study period. There were significant differences between subgroups in both number- and days of hospitalization pre study (p = 0 0.004 and 0.003, respectively) and in study (p< 0.001 and p = 0.01). A significant increase in number of hospitalizations was seen in the subgroup with 0–1 exacerbation pre study inclusion (p = 0.01) as well as a close to significant increase in days of hospitalization (p = 0.08). Performing one-sample t-test, comparing with the mean at baseline, the p-value was similar for number of admissions (p = 0.01) and even closer to significant (p = 0.052) for days of admission. A significant reduction in number of both number- and days of hospitalizations were seen in the subgroup with 2+ exacerbations pre study inclusion (p = 0.002, respectively 0.025).

The supplementary information provides a comparison of change in exacerbations; number and days of admission between change in number of exacerbations; days- and number of hospitalization pre- and in study in patients with 0–1 exacerbations and controls from the former RCT-study [17] (S1 and S2 Figs). There was no statistical difference between groups.

When exploring potential influence of other parameters on number of exacerbations in study in the entire study population, correcting for days of treatment with HFNC, there was a significant correlation between exacerbations and both number- and days of admission pre study and a reduction in number of exacerbations in study (p<0.001), corrected for time of use of HFNC. No correlation was seen between PaCO_2_ (p = 0.09) on number of exacerbations in study at baseline. However, in the subgroup 0–1 AECOPD there was no significant correlation between exacerbations, admission number- and days pre study to exacerbations in study (p = 0.08, p = 0.1 and p = 0.08 respectively), whereas in the subgroup with 2+ AECOPD pre study the correlation remained significant (p = 0.001). There was no correlation to PaCO_2_ in either of the analyses.

A significant correlation was seen between number of hospitalizations pre study and number of hospitalizations in study when correcting for days of treatment with HFNC; number of exacerbations and days of hospitalization 12 months pre study in the entire study population (p = 0.01). When repeating the analysis above in the subgroups with 0–1 exacerbation the year pre study no significant correlation was seen (p = 0.48) whereas in the subgroup with 2+ exacerbations 12 months prior to study entry the correlation was even stronger (p<0.001).

A significant correlation also was seen between days of hospitalizations pre study; number of AECOPD as well as days of hospitalization 12 months pre study and days of hospitalizations in study in the entire study population when correcting for days of treatment with HFNC (p = 0.02). Repeating the analysis above in the subgroups with 0–1 exacerbation the year pre study no significant correlation was seen (p = 0.65) whereas in the subgroup with 2+ exacerbations 12 months prior to study entry the correlation was even stronger (p<0.001).

## Discussion

This study indicates a reduction in number of exacerbations; number- and days of hospitalizations in patients with COPD and chronic hypoxic failure, treated with HFNC adjacent to LTOT, with two or more exacerbations in the year prior to inclusion in the study. Furthermore, that consistent use of HFNC may be beneficial to these patients.

This study suggests HFNC to be a beneficial add on for reduction of exacerbations in patients with COPD, chronic hypoxic failure and frequent exacerbations. The beneficial effect was also shown in a previous paper on this study population [12], and this post-hoc study narrows in the patients with special benefit of the treatment. Personalized medicine is in general the recommendation of today in COPD [5]. Nonetheless, in the complex group of COPD patients with chronic hypoxic failure this remains complicated, although a number of treatment options have in recent years been shown effective in preventing exacerbations in this patient category, such as rehabilitation and pharmacotherapy and home mechanical ventilation (HMV) [18–20]. The HoT-HMW study showed beneficial effect on number of exacerbations in COPD patients with hypercapnia [19], unfortunately the treatment option is not well tolerated by all patients. A recent short-term study by Bräunlich and colleagues indicated equality in reduction of PaCO_2_ and improvement in quality of life between the two treatment modalities, however long-term studies are needed to compare the effect of HMV and HFNC on exacerbations. Still, this post hoc analysis contributes to the emerging pool of knowledge that is needed for future recommendations for personalized medicine in patients with COPD, chronic hypoxic failure and frequent exacerbations.

In line with the above, the Aalborg study previously showed a reduction in number of hospitalizations when correcting for use of HFNC [12]. This study shows that in patients with two or more exacerbations HFNC reduces both number- and days of hospitalizations, even when looking at the uncorrected data, but even more so when correcting for days of HFNC treatment. As above, studies in patients with advanced COPD investigating treatment options preventing hospitalizations are few. The HoT-HMV-study found beneficial effect of home mechanical ventilation (HMV) on admission and mortality in patients with COPD, chronic hypoxic failure and hypercapnia[19]. In the present study the inclusion criteria was only chronic hypoxic failure. As such, the two treatment modalities may be beneficial to different subgroups of patients or indications may change with disease progression. Katajisto et al recently showed rehabilitation to be preventive of hospitalization in patients with advanced COPD [21]. As for exacerbations, future studies will have to investigate the place of the different preventive strategies in COPD patients in need of LTOT.

In this study both number of exacerbations and hospitalizations increased in patients with 0–1 exacerbations in the year prior to inclusion. The two subgroups analyzed here thereby somehow contradict each other. Does that then mean that HFNC is harmful to some subgroups of patients? In the RCT from which this post-hoc study emerges, an increase in both number of exacerbations and hospitalizations were seen in controls, similar to that of patients with 0–1 exacerbations in the previous year [12]. From the figures in the appendix it is also evident that patients with 0–1 exacerbation pre study merely behaves as comparable patients in the background population with the same severity of disease does. This is consistent with what has been described previously as the natural history of COPD where the disease progress, despite the use of treatment options to delay the disease development [22]. As such, HFNC is not harmful, yet may not be useful in terms of reducing number of exacerbations and hospital admissions. However, HFNC may be of benefit to patients with 0–1 exacerbations in terms of I.e. reduction in dyspnea; ameliorations in quality of life and other secondary endpoints of the RCT, however this is not within the scope of this study to investigate this.

There are a number of limitations in this post hoc analysis. Numbers in the subgroups are small; however, the post hoc analysis was adequately powered to investigate significant changes in number of exacerbations in the groups with 2+ exacerbations at baseline. Therefore, the significant findings do provide us with an indication of a subgroup that may benefit from domiciliary high-flow treatment. Looking at the comparison to controls in the appendix there are indications that patients with 2+ exacerbations pre-study have been the ones that have generated the significant findings in the original RCT. As domiciliary high-flow treatment in COPD still is in its cradle, we do need these indications to design our future studies. Therefore, the results of this study are therefore not conclusive, merely suggestive and should be used for scientific purposes than for recommendations. Secondly, we do not know whether HFNC is equally effective in mild and severe exacerbations, as exacerbation severity was not registered in the original RCT. However, as all hospitalizations included in the analysis were COPD related, we do know that HFNC has an effect on COPD patients with chronic hypoxic failure and frequent exacerbations, and by the reduction of exacerbations described in this study, we know that it has an effect on mild and moderate AECOPD. However, future studies should include further characteristics of the exacerbations to address this in detail. Secondly, as HFNC days were used and not hours per day, we still do not know whether the diurnal pattern of use influence outcome. Readings on hours of use were made every fortnight; however, we do not know the patients’ exact pattern of use of the device. We do know, however, that the less patients actually used HFNC, in terms of hours of use, read from the device, the more they over-reported the use of HFNC [23]. As such, the validity hours reported by the patients were not sufficient for the parameter to be included in this study. As such, this is a matter for future technical development of the device and future studies. Lastly; exacerbations prior to inclusion in this study were self-reported and therefore may be biased. However, the distribution in the control-group in in the initial RCT was similar to the one in the high-flow treated group and similar to the distribution comparable COPD-patients in the ECLIPSE-cohort [24].

In conclusion, in patients with COPD and chronic hypoxic failure those with two or more exacerbations in the preceding year may benefit from the use of HFNC, in terms of reducing exacerbations and hospitalization. However, larger studies should confirm these findings.

## Supporting information

S1 Fig Description of exacerbations, number- and days of hospitalisation pre- and in study (N) for controls; patients with 0–1 exacerbation pre study and patients with 2+ exacerbations pre study. (TIF)

S2 Fig Change (Δ) in number (N) of exacerbations, number and days of hospital admissions in controls and patients with 0–1 exacerbation pre study. No significant differences were seen between controls and patients with 0–1 exacerbations pre study, as demonstrated in the figure, whereas significant changes were seen between patients with 2+ exacerbations pre study and controls in Δ exacerbations (p = 0.01); Δ hospital admission (p = 0.03) and Δ admission days (p<0.0001).
(TIF)
