# Supplementary figures and images for: Domiciliary high-flow treatment in patients with COPD and chronic hypoxic failure: In whom can we reduce exacerbations and hospitalizations?
Source: PLoS One. 2019 Dec 30;14(12):e0227221. doi: 10.1371/journal.pone.0227221 (PMC6937157; doi:10.1371/journal.pone.0227221)

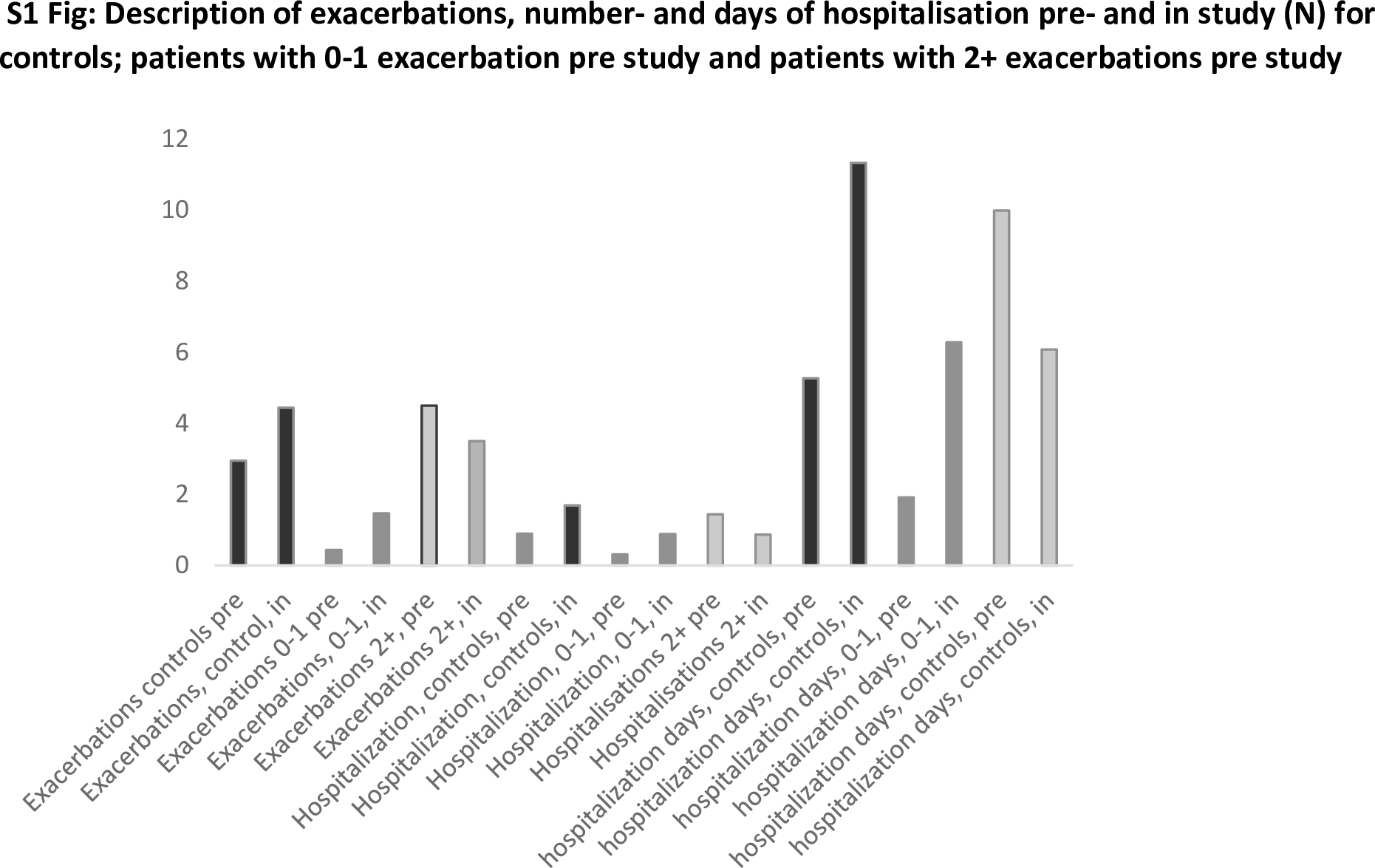

Supplement: S1 Fig — (TIF) [file pone.0227221.s001.tif]

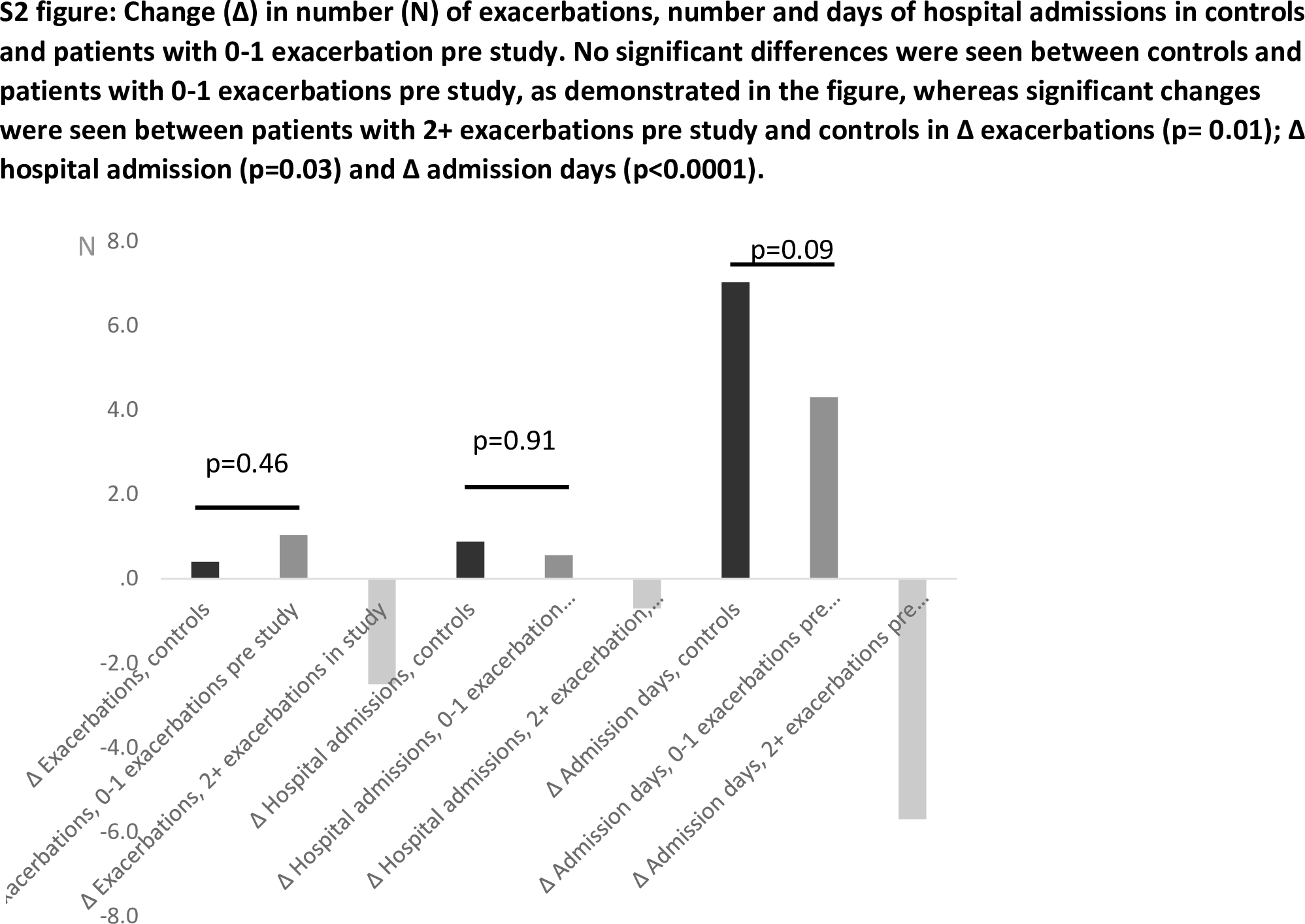

Supplement: S2 Fig — No significant differences were seen between controls and patients with 0–1 exacerbations pre study, as demonstrated in the figure, whereas significant changes were seen between patients with 2+ exacerbations pre study and controls in Δ exacerbations (p = 0.01); Δ hospital admission (p = 0.03) and Δ admission days (p<0.0001). (TIF) [file pone.0227221.s002.tif]
